# The basal transcription machinery as a target for cancer therapy

**DOI:** 10.1186/1475-2867-14-18

**Published:** 2014-02-28

**Authors:** Claudia Villicaña, Grisel Cruz, Mario Zurita

**Affiliations:** 1 Departament of Developmental Genetics, Instituto de Biotecnología, Universidad Nacional Autónoma de México, Mexico, Mexico

**Keywords:** Transcription inhibition, Cancer therapy, Gene expression, RNA polymerase

## Abstract

General transcription is required for the growth and survival of all living cells. However, tumor cells require extraordinary levels of transcription, including the transcription of ribosomal RNA genes by RNA polymerase I (RNPI) and mRNA by RNA polymerase II (RNPII). In fact, cancer cells have mutations that directly enhance transcription and are frequently required for cancer transformation. For example, the recent discovery that MYC enhances the transcription of the majority genes in the genome correlates with the fact that several transcription interfering drugs preferentially kill cancer cells. In recent years, advances in the mechanistic studies of the basal transcription machinery and the discovery of drugs that interfere with multiple components of transcription are being used to combat cancer. For example, drugs such as triptolide that targets the general transcription factors TFIIH and JQ1 to inhibit BRD4 are administered to target the high proliferative rate of cancer cells. Given the importance of finding new strategies to preferentially sensitize tumor cells, this review primarily focuses on several transcription inhibitory drugs to demonstrate that the basal transcription machinery constitutes a potential target for the design of novel cancer drugs. We highlight the drugs’ mechanisms for interfering with tumor cell survival, their importance in cancer treatment and the challenges of clinical application.

## Review

Cells require transcription for basic processes, such as survival, cell growth and differentiation. Transformation highly correlates with enhanced transcription of oncogenes and other transcription factors in cancer cells [1]. For many years, genotoxic drugs have been administered to combat cancer. In fact, several compounds that reduce cancer cell proliferation also directly or indirectly affect global transcription, a characteristic that mechanistically contributes to their cytotoxicity. For example, intercalator compounds, such as cisplatin, induce DNA damage but also disrupt transcription [1,2].

Interestingly, several studies in various models have demonstrated that oncogenically transformed cells are more susceptible to apoptosis in response to transcriptional inhibition, suggesting that transcriptional inhibition has direct cytotoxic effects in malignant cells. In mouse lymphoma models, tumor cells were more sensitive to apoptosis than wild-type cells after treatment with an inhibitor of RNPI (RNA polymerase I) transcription [3], and similar effects were observed using RNPII (RNA polymerase II) inhibitors [4-6]. The reduction of basal transcription may interfere with transcriptional programming directed by key oncogenes, thereby exerting a greater effect in cancer cells versus normal cells. These findings support the link between transcription and transformation, suggesting that the basal transcription machinery is a promising druggable target to block cancer cell proliferation. Thus, the basal transcription machinery is an increasingly important target for cancer therapies.

In this review, we briefly describe transcriptional machinery components and several transcription inhibitory drugs. We discuss how these drugs interfere with survival of tumor cells as well as the challenges and limitations for clinical application.

### General transcription machinery: RNPI and RNPII

**Figure 1:**
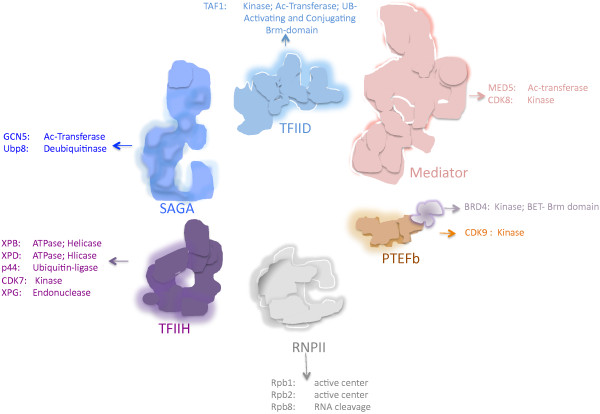
**Different subunits of protein complexes involved in basal in basal transcription have different enzymatic activities that are or can be target by drugs inhibiting RNPII transcription.** In the case of BRD4, in addition to its kinase activity, the Brm domain is the target of JQ1 and I-BET151, which interferes with the binding to acetylated histones; therefore future drugs that target components of the basal transcription machinery could be designed to interfere the interaction between different subunits into the complex. The different subunits with enzymatic activities of a corresponding complex are indicated in the figure.

The basal transcription machinery, a central component of general transcription, consists of several universal components that are required for promoter interaction, thereby achieving efficient and regulated transcription. Several components of these transcriptional complexes exhibit enzymatic activity, which can potentially be inhibited by newly designed drugs (Figure 1). In addition, other drugs can be designed that disturb the protein-protein or DNA-protein interactions of these complexes.

#### RNPI-mediated transcription

The rRNA genes are located in the nucleolus and are transcribed by RNPI in eukaryotic cells. rRNA genes are transcribed during the S and G2 phases of the cell cycle; however, transcription is repressed during mitosis and is slowly recovered in G1 [7]. Selectivity factor 1 (SL1 in humans, TIF-1B in mouse) and upstream binding factor (UBF) are required for promoter binding and the recruitment of RNPI as well as various accessory factors, such as TFIIH; protein kinase CK2; nuclear actin; myosin (NM1); the chromatin modifiers G9A, PCAF and SIRT7; proteins involved in replication and repair; oncogenes and tumor suppressors for review, see: [7,8]. Moreover, rRNA gene expression is highly regulated in response to various environment conditions, including growth factors, nutrients and stress. Thus, the modulation of rRNA synthesis involves several signaling pathways that regulate cell growth and proliferation [8].

#### RNPII-mediated transcription

The main component of the basal transcription machinery is RNPII, which is composed of 12 subunits [9] and requires accessory factors, such as TFIIA, TFIIB, TFIIE, TFIIF, TFIID, TFIIH, SAGA, P-TEFb and Mediator, at various steps of transcription for review, see [9,10].

TFIID and SAGA (Spt–Ada–Gcn5–acetyltransferase) interact with the promoter and participate in the recruitment of the pre-initiation complex (PIC). SAGA specifically activates gene transcription in response to environmental stress [11-13]. After SAGA or TFIID binding to the promoter, several transcription factors are recruited to form the PIC. PIC factors include TFIIA, TFIIB, TFIID, TFIIE, TFIIF, TFIIH, Mediator and RNPII [10]. TFIIH not only participates in transcription but also nucleotide excision repair (NER) and cell cycle regulation. The XPB subunit is important for open complex formation, which is critical for promoter escape. CDK7 phosphorylates Ser 5 and 7 in the carboxy-terminal domain (CTD) heptapeptide repeat sequence of the large subunit of RNPII, which is important for the recruitment of the mRNA processing machinery during transcription [14,15].

Mediator is a multisubunit complex that cooperatively binds with RNPII and a subset of general factors during an intermediate step of PIC formation. Mediator is a transducer of the signals that link the activators with the general transcription machinery, thereby activating transcription [16,17]. However, these signals inhibit RNPII in certain scenarios [18,19]. On the other hand, the positive elongation factor (P-TEFb) complex is fundamental for transcription elongation through RNPII. The CDK9 subunit of P-TEFb phosphorylates the CTD of RNPII at Ser 2 for transcription elongation [20,21]. Interestingly, P-TEFb interacts with the super elongation complex (SEC) [22]. In addition, P-TEFb also interacts with the bromodomain proteins BRD3 and BRD4, which are required for the efficient recruitment of the P-TEFb complex to the promoter and the activation of transcription elongation through binding to acetylated histones [23,24]. Recently, it was demonstrated that BRD4 is a kinase that phosphorylates Ser 2 of the RNPII CTD domain, suggesting a direct role for BRD4 in transcription elongation [25].

### Cancer cells require high levels of transcription

**Figure 2:**
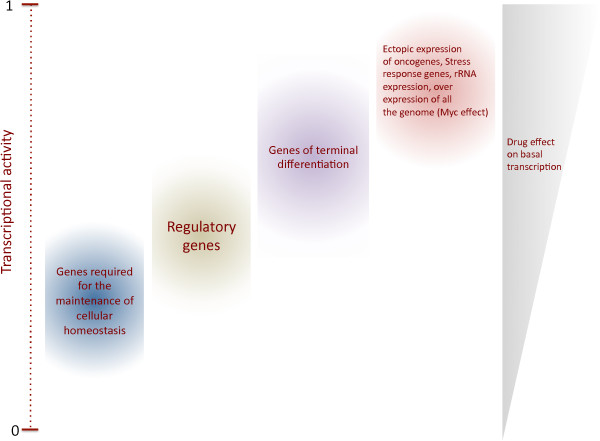
**Cancer cells require high levels of basal transcription.** To maintain a proliferative state cancer cells need active transcription by the three RNA polymerases. In particular the expression of oncogenes as well genes that suppress apoptosis is enhanced in tumour cells. Also, the enhancement of global transcription by MYC is necessary to maintain the cancerous phenotype. This situation is similar to the requirements of the transcription activity in ectopic expression genes, which is more sensible to the reduction of global transcription than normally expressed genes. Different kind of genes in the genome, require different levels of transcriptional activity. For instance, metabolic and regulatory genes do not require high levels of transcriptional activity. On the other hand, genes that express product of terminal differentiation require higher levels of transcriptional activity. Unregulated and ectopically expressed genes as well as overexpressed genes as response to stress, a situation that occurs in many cancers, require even higher transcriptional activity to maintain a transformed phenotype. Therefore, the reduction of the basal transcription activity preferentially affects these genes.

Transformed cells require active transcription for proliferation and survival. Certain oncogenes, ribosomal genes and components of the transcriptional machinery are overexpressed in tumor cells to maintain proliferation [8,26,27]. For RNPI transcription, increased rRNA synthesis is associated with uncontrolled cancer cell proliferation. In fact, enhanced RNPI activity triggers nucleoli enlargement, a marker of aggressive cancer cells associated with a poor prognosis [28]. In addition, RNPII transcription is required to support the high demand of the transcripts, including oncogenes and anti-apoptotic factors, which is necessary for the maintenance of rapid growth and apoptosis resistance (Figure 2). For example, RNPII increases the global transcription of the majority of genes expressed in the cell through P-TEFb, which is important for maintaining the transformed phenotype [29,30]. Likewise, RNPIII activity is increased in tumor cells compared with normal cells, and the overexpression of BRF2, a RNPIII factor, is associated with several cancers [27].

Interestingly, the depletion of Xist RNA, involved in mammalian X-chromosome inactivation, induces hematological cancer in mice due to the increased expression of X-specific transcripts that are potentially associated with carcinogenesis [31]. In this work, Xist RNA depletion induces genome-wide alterations given that X-chromosome reactivation enhances transcription of X-specific transcripts, suggesting that Xist RNA is a cancer suppressor *in vivo*. Additionally, cancer cells can modulate their transcriptome and physiology to enhance survival and proliferation under stress conditions. This notion was demonstrated for heat shock factor 1 (HSF1), which drives a transcriptional program different from the heat shock supporting oncogenic processes, including protein folding, stress response, cell cycle and signaling genes, to maintain highly malignant human cancers [32]. These findings highlight the important role of transcription in maintaining the transcript supply of cancer cells.

### Transcription inhibitors

Drugs that potentially target the basal transcription machinery components could preferentially affect highly proliferative cells. Various components targeted by drugs include cyclin-dependent kinases (CDKs), RNA polymerases or components of associated transcriptional complexes.

#### CDK and kinases

Several CDKs that participate in RNPII transcription are targets for global transcription inhibition. CDK7 is a component of the basal transcription factor TFIIH that phosphorylates Ser 5 and 7 in the C-terminal domain (CTD) heptapeptide repeat sequence of the large subunit of RNPII, which is important for promoter escape and the recruitment of the mRNA processing machinery during transcription. CDK7 is a target of global transcription inhibition [14,15]. Additionally, CDK9, a component of P-TEFb, phosphorylates the CTD of RNPII at Ser 2 for transcription elongation [20,21]. Thus, many drugs target CDKs given that CDKs are deregulated in cancer cells. Mechanistically, CDK inhibitors compete with ATP at the enzyme active site. Therefore, CDK inhibition results in RNPII hypophosphorylation [33]. Although CDK inhibitors affect the activity of CDK7, CDK8 and CDK9, most drugs have affinities for several targets, including CDKs not related to transcription and other kinases (see Table 1) [34].

**Table 1:** Drugs targeting proteins involved in transcription

**Drug**	**Target**	**Mechanism of action**	**Class gene inhibited**	**Other targets**	**Cancer treatment**	**References**
H-7	CDK7, CDK8, CDK9	Reduce levels of phosphorylated RNP lII inhibiting elongation	I, II	PKC	Only research	[35-38]
H-8	CDK7, CDK9, CDK8	Reduce levels of phosphorylated RNP II inhibiting elongation	I, II	PKA, PKC, PKG, MLCK	Only research	[29,30]
AT8319	CDK9	Inhibits RNP II phosphorylation on Ser 2 disrupting transcription elongation	II	ND	MM, advanced solid tumors, and refractory non-Hodgkin’s lymphoma	[34]
Dinaciclib/ SCH-727965	CDK9	Inhibits RNP II phosphorylation on Ser 2 disrupting transcription elongation. Impaired rRNA processing	I, II	CDK1, CDK2, CDK4, CDK5, CDK7	Solid tumors, hematologicalmalignancies, MM, melanoma, plasma cell neoplasia	[33,34]
RGB-286638	CDK9	Inhibits Ser 2 phosphorylation of RNP II disrupting transcription elongation	II	CDK1, CDK2, CDK4, CDK5, CDK6, CDK7	Hematological malignancies	[34]
R547	CDK9	Inhibits Ser 2 phosphorylation of RNP II disrupting transcription elongation	II	CDK1, CDK2, CDK4, CDK5, CDK7	Solid tumors	[34,39]
P276-00	CDK9	Inhibits transcription elongation	II	CDK1, CDK4	MM, breast, pancreas, melanoma, MCL, HNSCC	[34]
DRB	CDK9	Inhibits RNP II phosphorylation on Ser 2. Impaired rRNA processing	I, II	CDK2, CDK4, CDK7, CDK8, casein kinase I and II	Only Research	[33,34,40]
Roscovitine/ Seliciclib	CDK7 and CDK9	Acts as a competitor for ATP binding inhibiting kinase activity and Ser 5 phosphorylation or RNP II Inhibits rRNA processing	I, II	Cdc2, CDK2, CDK5, Erk1, Erk2, Dyrk, piridoxal kinase	Breast, solid tumors, B-cell malignancies, non-small cell lung cancer, and nasopharyngeal cancer	[33,41-43]
ARC	CDK9	Inhibits phosphorylation Ser 2 and Ser 5 of RNP II inhibiting transcription elongation	II	PKC	CLL, ALL, hairy cell leukaemia	[5,44]
ZK 304709	CDK7, CDK9	Inhibits RNP II phosphorylation on Ser 2.	II	CDK1, CDK2, CDK4, VEGFR1-3, PDGFR-β, Flt-3	Relapsed and/or refractory tumors	[45]
Wogonin	CDK9	Inhibits RNP II phosphorylation on Ser 2.	II	CDK7	Xenografts	[46]
CDKI-71	CDK9	Inhibits RNP II phosphorylation on Ser 2.	II	CDK1, CDK2, CDK7, CDK6	Under evaluation in cancer cell lines	[47]
Flavopiridol	CDK9, CDK8	Inhibits phosphorylation of Ser 2 in CTD of RNPII and interrupts RNA elongation; impaired rRNA processing	I, II	CDK1, CDK2, CDK4, CDK6, CDK7, PKC, Src, EGFR, ERK1	CLL, MM, MCL, indolent B-cell non-Hodgkin’s lymphomas, germ line tumor, melanoma, ALM	[33-35,48]
SNS-032	CDK9	Inhibits Ser 2 phosphorylation of RNP II disrupting transcription elongation	II	CDK2, CDK7, GSK3	CLL, ALL, MM	[34,45]
AT7519	CDK9	Inhibit RNP II phosphorylation of Ser 2 and 5	II	CDK2, CDK4, CDK5, GSK-3	MM, solid tumor	[34,45,49]
CX-5461	SL1 complex	Disrupts formation of SL1-rDNA complex	I	ND	Lymphoma and leukemia human cancer xenograft model	[3, 671]
α-amatinin	RNP II and III	Binds to the largest subunit of RNP II and RNP III	II, III	ND	None due to hepatotoxicity	[33,50]
TAS-106	RNA polymerases	Ribonucleoside Inhibits RNA polymerases	I, II and III	ND	Solid tumors	[51,52]
Triptolide	XPB subunit of TFIIH	Inhibits RNP I and II by inhibiting XPB ATPase activity. It triggers RNP II degradation	I, II	Polycystin-2 calcium channel, ADAM10.	Leukemia, myeloma, lymphoma, cholangiosarcoma, hepatocelular, cervical, pancreatic, gastric and oral cancer, anaplastic thyroid carcinoma	[33,39,53-55]
BMH-21	RNA polymerase I	Degradation of the RPA194 subunit of the RNA polymerase I	I	Induce p53	Melanoma	[56]
	XPB subunit of TFIIH	Promotes XPB degradation	II	Antagonist of aldosterone	Sensitizes carcinoma cells to cis- platinium	[57]
JQ1 and I-BET151	BRD3 and BRD4	Displace BRD3 and BRD4 from chromatin	II	ND	Multiple myeloma, leukaemia, lymphoma and lung adenocarcinoma in animal models	[57-63]

*Abbreviations*: ND, non detected; CLL, chronic lymphocytic leukemia; ALL, acute lymphocytic leukemia; AML, acute myeloid leukemia, CML, chronic myelogenousleukemia; HNSCC, head and neck squamous cell carcinoma; MCL, mantle cell lymphoma; MM, multiple myeloma.

The most commonly used transcription inhibition drugs are flavopiridol and DRB. The flavone flavopiridol is a potent CDK9 inhibitor with a *k*_*i*_ of 3 nM. In addition, flavopiridol inhibits CDK8 with a *k*_*i*_ of 18 nM [35,64]. In chronic lymphocytic leukemia (CLL), flavopiridol cytotoxicity is associated with transcription inhibition mediated by the anti-apoptotic factor Mcl-1 [64]. In addition, flavopiridol is highly toxic, causing severe side effects and acute lysis tumor syndrome [34,65]. In fact, it was recently reported that flavopiridol induces double strand breaks (DSBs), explaining the drug’s toxicity [47]. DRB (5,6-dichloro-1-β-D-ribofuranosylbenzimidazole) is an adenosine analog that specifically inhibits CDK9 and moderately inhibits CDK7 and CDK8, disrupting initiation and elongation [33,66]. DRB is widely used as a transcription inhibitor and to measure transcription rates given its rapid uptake; however, it is not used to treat cancer [33].

The isoquinolinesulphonamide derivatives H-7 and H-8 inhibit transcription elongation, inducing RNPII dephosphorylation [36]. However, these compounds belong to a broad spectrum of protein kinase inhibitors that target other kinases, such as PKC, PKA and PKG, with affinities similar those reported for CDK7, CDK8 and CDK9 [35,67]. Isoquinoline compounds are widely used to inhibit signaling pathways to elucidate signal transduction mechanisms; these compounds are typically not employed for cancer treatment [68].

Other CDK inhibitors exhibit cytotoxicity due to the transcriptional inhibition of anti-apoptotic proteins [34]. A number of these drugs are currently approved for cancer treatment, and others are currently being explored in clinical trials for various malignancies (Table 1). Transcription inhibitors can also downregulate genes involved in angiogenesis and metastasis. For example, the anti-angiogenic properties of flavopiridol and SNS-032 are partially attributed to the downregulation of VEGF mRNA and protein, the most potent tumor angiogenic factor [69-71].

Other novel compounds exhibit promising antitumor activity and display lower toxicities compared with traditional inhibitors. CDKI-71 is a novel CDK9 inhibitor that displays a high affinity for CDK9 (*k*_*i*_?=?6 nM), similar to flavopiridol. In fact, CDKI-71 induces apoptosis by downregulating the anti-apoptotic factor Mcl-1 in various cancer cell lines with minimal effects in normal fibroblasts and B and T-cells. Interestingly, CDKI-71 triggers apoptosis in several cancer cell lines with heterogeneous genetic backgrounds, including Rb and p53 mutations, suggesting that cell death is p53-independent [47]. Given that several tumors harbor p53 mutations, the activation of p53-independent cell death pathways represents a promising option to induce apoptosis in these tumors. Ibulocydine is a CDK inhibitor pro-drug that targets CDK7 and CDK9, thereby triggering apoptosis. The apoptotic effects of ibulocydine result in the downregulation of expression of anti-apoptotic factors, including Mcl-1, XIAP and survivin. Ibulocydine is currently under investigation and has exhibited promising results. For example, ibulocydine induces apoptosis without toxic side effects in mouse xenografts of hepatocellular carcinoma (HCC) [72].

Other compounds, such as hypericin, rottlerin and SP600125, are kinase inhibitors that inhibit transcription. The mechanisms of action for each drug have not been described. However, these drugs inhibit TBP phosphorylation during elongation, and SP600125 also inhibits phosphorylation at Ser 2 and 5 of the CTD of RNPII [73]. Similar to other drugs, these compounds also have additional targets. Hypericin inhibits epidermal growth factor, PKC and MAP kinase [74,75], whereas rottlerin inhibits PRAK and MAPKAP-K2 [76]. SP600125 inhibits JNK [77]. These drugs only have been characterized at the biochemical level; thus, research involving *in vivo* models should be explored. Wogonin, a flavone isolated from *Scutellaria baicalensis*, was recently identified as a novel compound. Wogonin inhibits CDK9 and blocks the phosphorylation of the CTD of RNPII. Wogonin induces apoptosis and suppress growth in several cancer cell lines and human cancer xenografts, respectively. The antitumor properties of wogonin reduce RNA synthesis and downregulate Mcl-1 in a fashion that primarily affects malignant versus normal T-cells [46].

#### RNP enzymes

Although the expected targets for transcription inhibition are RNP enzymes, only a few drugs that directly affect these enzymes have been described. To date, α-amanitin and TAS-106 are two drugs that directly target RNP enzymes and inhibit transcription. α-amanitin is a cyclic octapeptide isolated from *Amanita* mushrooms that is extremely toxic. α-amanitin inhibits RNPII and III but not RNPI. RNPII is more sensitive to α-amanitin compared with RNPIII, which is a hundred-fold less sensitive than RNPII. The mechanism of action for α-amanitin involves binding to RNA polymerase to prevent DNA and RNA translocation, but α-amanitin does not affect nucleotide entry and RNA synthesis [33]. Although α-amanitin is an effective and specific transcription inhibitor, it is not used in cancer treatment due to high hepatotoxicity [50].

TAS-106 (1-(3-C-ethynyl-b-D-ribo-pentofuranosyl)cytosineECyd) is a cytidine analog that exhibits potent cytotoxic and anti-tumor properties against solid tumors. TAS-106’s main mechanism of cytotoxicity is inhibition of RNPI-, II- and III-mediated RNA synthesis, thereby inducing apoptosis [78]. TAS-106 reduces the transcription of several factors required for survival. For example, TAS-106 induces apoptosis in radiation-resistant solid tumor cells through the depletion of hypoxia-inducing factor (HIF-α) [79]. In addition, TAS-106 also triggers apoptosis in cancer cells by reducing DSBs repair via BRCA2 transcript depletion [51].

Recently, a study reported that BMH-21, a compound that is a potent p53 activator and DNA intercalator at GC rich regions, which are abundant in the rRNA genes promoter, induces the degradation of the RPA194 subunit of RNPI, the largest RNPI subunit [56]. As a consequence, reduced rRNA synthesis generates a potent anticancer effect [56]. This effect is independent of p53 and opens the possibility that this drug may be used in cancer treatment. However, it is important to determine the effect of BHM-21 on other GC-rich regions in the genome, such as GpC islands.

#### Associated transcriptional complexes

Transcription can be disrupted via targeting of associated transcriptional complex components. Triptolide is a diterpene triepoxide that covalently binds to the XPB subunit of TFIIH and inhibits its ATPase activity. This action disrupts the opening of double-stranded DNA for RNPII transcription and repair as well as RNPI transcription [53,80-82]. In fact, triptolide cytotoxicity is associated with the transcriptional inhibition of anti-apoptotic factors and the induction of apoptotic factors [83]. Triptolide has been widely used for the treatment of various cancers with promising outcomes (See Table 1). In *Drosophila*, triptolide phenocopies mutations in TFIIH subunits, inducing apoptosis in the wing discs. Interestingly, apoptosis is enhanced in p53-deficient cells by JNK pathway activation [84]. This finding is interesting as the majority of solid tumors harbor mutations in p53 or components of the p53 pathway, suggesting that triptolide potentially induces apoptosis via JNK in these malignancies. In addition, TFIIH also participates in RNPI-mediated transcription, and triptolide inhibits RNPI elongation [54]. Therefore, triptolide not only affects TFIIH in RNPII transcription but also affects rRNA synthesis. With regard to the XPB subunit of TFIIH, a recent small molecule screen identified spironolactone (SP) as a compound that inhibits nucleotide excision repair (NER) [57]. Intriguingly, SP promotes XPB degradation via ubiquitination and the proteasome. Therefore, SP affects both NER and RNPII-mediated transcription. In this work, the authors focused on the role of SP in potentiating the effect of platinum in the induction of NER in cancer cells; however, SP is also a compound that may be used to treat cancer by inhibiting RNPII transcription, similar to triptolide.

BRD4 inhibition by JQ1 is an emerging and relevant target for the treatment of various cancers. JQ1 is a thieno-triazolo-1,4-diazepine that displaces BET bromodomains from chromatin through competitive binding to the acetyl-lysine recognition pocket, preventing BRD4 reader activity [58]. JQ1 reduces c-MYC or FOSL1 transcription in multiple myeloma, leukemia, lymphoma and lung adenocarcinoma models [59-61]. However, recent evidence indicates that BRD3 and BRD4 exhibit a more generalized effect on gene expression than previously suggested; therefore, the inhibition of these factors has a generalized effect on RNPII transcription [30]. In addition, the GSK12015A (I-BET151) inhibitor developed by GlaxoSmithKline (GSK) displaces BRD3 and BRD4 from chromatin. This action causes the downregulation of BCL-2, MYC and CDK6, thereby inducing cell cycle arrest and apoptosis in leukemia models [62].

CX-5461 is another potent and selective drug that inhibits rRNA transcription by targeting RNPI basal machinery. CX-5461 specifically inhibits rRNA synthesis by directly preventing the interaction between the SL1 complex and rDNA. CX-5461 does not affect RNPII transcription and DNA replication [85]. Interestingly, CX-5461 induces autophagic cell death in solid tumor cancer cells, whereas it induces apoptotic cell death in hematological malignancies [3,85]. Moreover, CX-5461 exhibits potent anti-tumor effects in murine xenografts [3].

### Significance of transcription inhibition in cancer treatment

One key of successful therapy is the identification of critical nodes in the oncogenic network that can be inhibited to promote tumor growth cessation by apoptosis, differentiation, necrosis or senescence. Several findings suggest that cancer cells require high transcription rates and harbor mutations or genetic backgrounds that favor enhanced transcription to sustain growth. Several MLL translocations and components of the SEC complex, including P-TEFb, AF9 and ELL, have been described in various leukemias, suggesting that transcription is misregulated in leukemias [22]. Likewise, CDK8 activity is also associated with the enhanced expression of genes regulated in response to serum and the Wnt/β-catenin pathway. In fact, CDK8 overexpression correlates with β-catenin deregulation in some colon cancers [86]. In addition, the importance of transcription inhibition is underscored by the notion that tumor cells are more sensitive to apoptosis than normal cells. This fact has been demonstrated in several models and tumor cell types [3-6]. Thus, the transcription machinery is an attractive putative target in cancer treatment because its specific tumor cell activity offers the possibility of directly attacking cancer cells and reducing the damage to healthy tissues.

The use of selective inhibitors for the transcriptional machinery offers advantages as these agents are considered less genotoxic to non-tumor cell populations compared with commonly used chemotherapy or radiation. This observation is important because a transcription inhibitor that specifically kills cancer cells may have a reduced incidence of secondary tumors compared with genotoxic agents that also affect normal cells. For example, chronic exposure to cisplatin leads to the development of resistance in patients and mouse models, and it has been demonstrated that enhanced damage repair contributes to tumor progression [87].

Initially, transcription-based therapies were primarily used to inhibit specific oncogenes or key genes required for tumor growth and survival. However, the use of these drugs was limited because the heterogeneity of some tumors allowed for the survival of cancer cell populations. For example, some tumors contain large hypoxic regions, which are generally are resistant to chemotherapy and radiation. Reduced MYC levels have been demonstrated in colon cancer cell lines. Reduced MYC expression is associated with survival because MYC is overexpressed and induces apoptosis in regions with poor energy supply [88]. In contrast, HIF is upregulated and contributes to survival in hypoxic tumors [89]. In these tumor conditions, exclusive MYC targeting is insufficient because each population exhibits different metabolic conditions; however, by targeting general transcription, highly expressed genes, such as MYC and HIF, can be repressed in various cell populations. Inhibitors can also repress transcription through the inhibition of kinases that regulate transcription factors or coactivators in specific tissues. For example, CDK9, which regulates the androgen receptor through direct phosphorylation and downregulation, decreases AR-transcription and proliferation genes in prostate cancer [90].

In addition, transcriptional inhibition also sensitizes stem cell populations that are generally chemoresistant. CDK8 and CDK9 are required for the maintenance of undifferentiated states of tumor embryonic stem cells [91,92]. CDK8 regulates the expression of a subset of genes involved in pluripotency. Moreover, CDK8 activity is partially mediated by MYC, indicating that additional independent mechanisms for the maintenance of undifferentiated states in tumors and stem cells exist. Thus, the loss of CDK8 induces differentiation. Similarly, JQ1-mediated BRD4 inhibition induces apoptosis in leukemic stem cells (LSC), which maintain and propagate the disease and are resistant to conventional chemotherapies [93]. Although JQ1 represents a powerful tool for the elimination of resistant cancer cells, its negative effects on the normal stem cell population must be evaluated to determine toxicity.

On the other hand, the lack of knowledge regarding the regulation of certain genes could generate different outcomes for the same treatment in clinical trials. For example, MYC is overexpressed in several tumors; however, MYC is surprisingly involved in the repression of integrins, which are required for breast cancer cell invasion and motility. This finding suggests that MYC inhibition is contraindicated in certain tumors [47]. A gene can possess oncogene or suppressor functions dependent on the context.

Regardless of the nature of tumorigenic signal, the transcription of certain oncogenic events can be disrupted by transcription inhibition, and therefore the transcriptional consequences cannot be predicted. Several recent findings suggest mechanisms involved in sensitization, which will be described in the following section.

#### Mechanisms of sensitization

Normal and tumor cells require transcription for survival. So, how does transcription inhibition lead to apoptosis in cancer cells? Transcription inhibition induces apoptosis by four possible mechanisms: altering the balance of apoptotic and anti-apoptotic factors to favor apoptosis, activating p53 and promoting its translocation to mitochondria, inhibiting DNA replication and promoting the accumulation of aberrant proteins in the nucleus [94]. Supporting the first mechanism, the mRNAs of many anti-apoptotic factors have short half-lives. The transcripts of oncogenes and regulators of proliferation also have reduced half-lives. Thus, although transcription inhibitors affect global mRNAs synthesis, oncogene transcripts may be more rapidly downregulated than other transcripts due to the rapid turnover of these mRNAs. Moreover, transcription inhibitors potentially interrupt the transcriptional program directed by these oncogenes, thereby disrupting the tumor’s physiological state [95].

**Figure 3:**
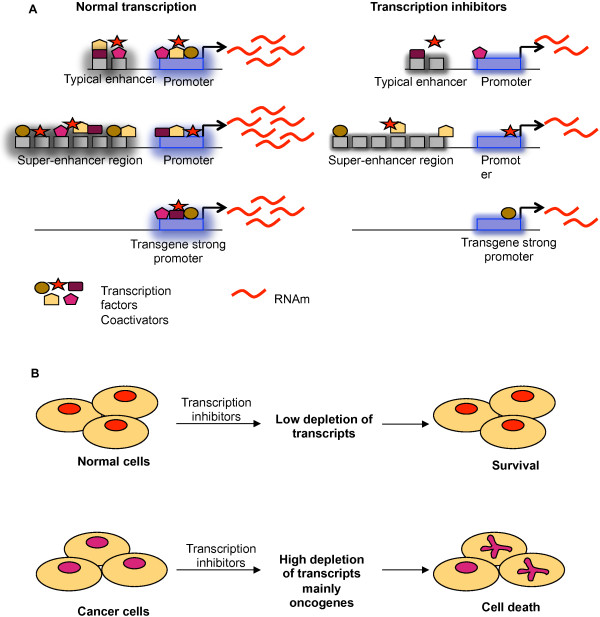
**Transcription inhibitors affect differentially regulatory sequences of genes and trigger cell death in cancer cells. A)** Transcription inhibitors differentially affect the regulatory sequences of genes and trigger cell death in cancer cells. This fact is related to requirement of transcription factors or coactivators recruited on regulatory sequences such as enhancers and promoters. Typical enhancers recruit several transcription factors and coactivators in order to enhance transcription of downstream gene; however, super-enhancer needs an excess of transcription than a typical enhancer, rendering the gene very sensitive to transcription perturbation. Similarly, transgene overexpression (whose promoter contain specific sequences for transactivators or specific *cis* elements), has been demonstrated to be affected by transcription inhibitors o genetic deficient in transcription [94,95]. In all cases, the final result is the depletion of messenger RNAs mainly genes that showed a high level of expression such as oncogenes. **B)** Cancer cells are more sensitive to suffer cell death after exposition to transcription inhibitors compared with normal cells. As we have seen before, transcription inhibitors cause a depletion of messenger RNAs mainly oncogenes and overexpressed genes; however, because cancer cells are oncogene-dependent for survival, their depletion triggers cell death preferentially in cancer cells, while preserving normal cells. This principle represents a strategic point for designing drug that targets directly cancer cells.

In *Drosophila*, the ectopic expression of various genes can be suppressed in a background that is partially deficient in transcription or by the pharmacological inhibition of transcription without affecting the proper expression of host genes [96,97]. Similar to this artificial overexpression system, oncogenes that are frequently overexpressed in cancer cells can be suppressed via transcription inhibition without affecting other genes (Figure 3). For example, various transcriptional inhibitors induce the downregulation of anti-apoptotic proteins, such as Mcl-1, XIAP and survivin [34], or oncogenes, such as MYC, without disrupting the transcription of normal genes in cancer cell lines [53,59]. Additionally, triptolide induces apoptosis by downregulating Bcr-Abl in K562 cells [98] or MYC in non-small lung cancer cells [83]. In addition to oncogenes, the class of genes called non-oncogenes significantly contributes to tumor survival; however, non-oncogenes do not induce cell transformation. Several non-oncogenes have been identified in a variety of tumors, where they are generally overexpressed [99,100]. Transcription inhibition also can disrupt the overexpression of non-oncogenes that are essential for cancer cell survival. For example, Heat shock Factor 1 (HSF1) is involved in survival in several cancer cell lines. Cancer cells are more dependent on HSF1 than normal cells. HSF1 depletion only minimally impacts normal cell viability, whereas cancer cells are strongly affected by HSF1 depletion [101]. In fact, triptolide induces cell death in pancreatic cancer cells via the inhibition of heat shock proteins [102]. Thus, it is likely that HSF1 depletion in these cells results from the inhibition of general transcription.

How does an inhibitor of general transcription selectively exert its effects on certain genes? Studies suggest that the high levels of expression by transgenes or gain of function mutations are dependent on their promoter, and these proteins are affected when transcription is inhibited or compromised. Moreover, *cis* structures on DNA called super-enhancers have been identified in genes required for cell identity and involved in cancer [63,103,104] (Figure 3). Super-enhancers significantly increase the expression of associated genes compared with typical enhancers. Super-enhancers are large DNA regions primarily occupied by mediator and other coactivators; therefore, super-enhancers are particularly sensitive to transcriptional perturbations that disrupt the transcriptional activity of genes associated with them. Indeed, the expression of Oct 4, a gene that contains a super-enhancer, is reduced in embryonic stem cells (ESC), inducing the downregulation of Mediator subunits [103]. More importantly, super-enhancers also have been identified in oncogenes, such as MYC, in multiple myeloma (MM) cells, and JQ1-mediated BRD4 inhibition causes MYC downregulation [63]. Given that super-enhancers require several-fold more transcription factors than typical enhancers, these structures are exquisitely sensitive to transcription disruption. Thus, it is possible that transcriptional inhibition by several drugs preferentially triggers apoptosis in cancer cells that express more oncogenes than normal cells. Additionally, super-enhancers are not associated with housekeeping genes, and it is plausible that these genes are not downregulated after exposure to transcription inhibitors, such as JQ1, thereby generating a selective effect [103].

In summary, transcription inhibition can interrupt transcriptional programs directed by key oncogenes or disrupt favorable growth conditions associated with the overexpression of non-oncogenes that contribute to survival and tumor progression.

### Challenges and limitations

#### Differential sensitivity of cancer cell lines

Transcription inhibition potentiates apoptosis and other types of cell death in tumor cells. However, studies have demonstrated differential responses to various drugs in a variety of cell lines and tumors. This differential sensitization might be dependent on a variety of factors, such as genetic background, tissue type or tumor heterogeneity. Thus, drugs must be evaluated experimentally to determine clinical efficacy.

Genetic background is a key factor responsible for the variable outcomes of chemotherapy. Triptolide treatment in two prostate cancer cell lines revealed that LNCaP cells (androgen dependent) are more sensitive to triptolide-induced apoptosis than PC-3 cells (androgen independent) [105]. These results indicate that different mechanisms are responsible for triptolide-induced apoptosis and that the genetic background of one cell line is more sensitive than the other. In addition, specific tumor mutations could be responsible for the differential responses. For example, DRB does not induce apoptosis in colon carcinoma cell lines with mutant p53 [106]. In fact, cells with p53 mutations are more chemoresistant [107]. With regard to tumor heterogeneity, this finding importantly indicates that random mutations in different cell populations within a tumor respond differently to the same treatment, thereby generating treatment-resistant populations. It is important to determine which factors are more effective in cancer stem cells to avoid the generation of secondary tumors. Numerous leukemia cell lines show differential sensitivities based on particular aberrations and genetic backgrounds. Exposure to the BRD4 inhibitor JQ1 sensitizes several leukemia cell lines but not the BCR/ABL1-positive chronic myelogenous leukemia cell line K562 [108].

In addition to genetic background, differences in the chemotherapy responses are dependent on the tissue type. For example, CX5461-treated solid tumor cancer cell lines display autophagy and senescence, not apoptosis. In contrast, CX5461 induces p53-dependent apoptotic cell death in hematological malignancies. However, no correlation exists between nucleolar stress and p53 status in solid tumor cell lines, indicating that the stress response is p53-independent [62]. In hematopoietic tissues, such as B-cells, the induction of cell death is highly dependent on p53, and p53 mutations have been associated with poor prognosis in hematological malignancies [109]. In contrast, other tissues, such as keratinocytes, are highly resistant to p53-dependent cell death, demonstrating a clear difference between tissues [110].

#### Unselective targets

Another interesting point to consider is unselectivity. In this review, we describe the transcription inhibitory activities of many drugs, but only a few drugs exhibit specific effects on transcription given that most drugs target several proteins. For example, most CDK inhibitory drugs exhibit a high affinity for numerous CDKs that are not involved in transcription and proteins that are not cyclins. This unselectivity does not distinguish the target’s transcriptional activities from its additional functions. Unselectivity might cause side effects given the association with unspecific partners. Moreover, cytotoxicity may be induced by agents that generate DNA damage, such as flavopiridol [47]. Thus, the design of more specific drugs is a current challenge. However, structural information regarding the enzyme’s active site or domains increasingly aids in the development of small compounds with more specific inhibitory activity that disrupts protein-protein and/or DNA-protein interactions.

#### Limitations

The targeting of transcriptional machinery represents an interesting mechanism to downregulate the key oncogenes driving tumorigenesis. However, transcription is an essential process that occurs in all living cells. Therefore, this strategy has several limitations. Whole organisms are comprised of several tissues and cell populations with different sensitivities to transcriptional perturbation. Specifically, the BRD4 inhibitor JQ1 disrupts cell specific factors in ESCs involved in pluripotency [103]. Therefore, it is important to determine whether targeting the transcription machinery affects the pluripotency of normal stem cell populations. JQ1 induces cell cycle arrest and apoptosis in AML cells; however, JQ1 only induces cell cycle arrest in normal bone marrow cells even when higher doses are administered [93]. Likewise, JQ1 does not display myelosuppressive effects in several *in vivo* models [59], suggesting that the cytotoxic effects are specific to acute myeloid leukemia (AML) cells. Nonetheless, the evaluation of results from long-term drug exposure is important to determine potential side effects in animal models and clinical trials.

The downregulation of certain proteins involved in transcription results in tumor suppression. For example, the downregulation of the MED-19 subunit of mediator inhibits cell growth and migration in tongue cancer [111], and CDK8 downregulation induces tumor cell differentiation [92]. Thus, mediator represents a putative target for drug inhibition. Recent work has shown that knockdown of the MED-12 subunit confers drug resistance in cancer cell lines by activating transforming growth factor (TGF-βR2) [112]; the activation of TGF-βR2 appears to be a unique function of the MED-12 subunit because other subunits of mediator do not localize outside of the nucleus, suggesting a MED-12-independent function of mediator. In addition to transcription and repair, studies suggest that the XPD and XPB subunits of TFIIH also participate in the regulation of processes, such as chromosome segregation and mitotic spindle dynamic, respectively [113,114]. BRD4 participates in the regulation of Aurora B expression. Reduced Aurora B expression causes severe cytokinesis and abnormal centrosomes in keratinocytes and cancer cells, increasing genomic instability [115]. Thus, the targeted selection of certain components of the transcription machinery should be evaluated if inhibition disrupts essential processes independent of transcription, causing toxicity or side effects.

### Combined and personalized therapy

The efficacy of transcription inhibitory drugs can be enhanced when combined with other treatments. Triptolide has been combined with several agents to significantly increase the apoptosis in several tumors using lower doses of the agents without increasing the side effects of chemotherapy [39]. JQ1 combined with Ara-C results in antileukemic effects in several AML malignancies [93]. Various cancers are resistant to certain drugs, but combinations of drugs with varying mechanisms of action can synergize to increase apoptotic induction and sensitize a variety of tumors compared with individually administered drugs.

Gene expression profiling of tumors can aid in the identification of molecular signatures that determine several tumor characteristics, such as proliferation rate, differentiation and metastatic capacity. The technique can also identify a possible spectrum of drugs that are effective for tumor growth inhibition as well as specific drug doses. In addition, possible drug synergies and the effects of genetic variation on clinical outcomes in patients can be predicted. For example, the BCR/ABL1-positive chronic myelogenous leukemia line K562 is resistant to JQ1; however, it may be sensitive to triptolide because triptolide may have a more penetrant effect on global transcription than JQ1 [108].

## Conclusions

Cancer cells require increased levels of transcription for growth and survival compared with normal cells. Cancer cells are more susceptible to alterations in gene expression. Various drugs that act as transcription inhibitors have been traditionally used in cancer treatment given that they preferentially reduce the global transcription of tumor cells. Thus, transcription inhibitors can potentially target the proteins that comprise the transcription machinery. As such, the transcription machinery is considered an ideal target for the design and improvement of drugs given that cancer cells are exquisitely sensitive to transcription inhibition and selectively affected by these drugs.

CDKs are one of the most common targets, but additional proteins have been targeted. However, some of these drugs have an affinity for other targets not involved in transcription, thereby generating possible side effects. Although drugs that inhibit transcription have challenges in clinical application, these drugs can be improved based on new discoveries regarding their mechanisms of action and information from clinical trials.

As previously demonstrated, the clinical application of drugs has several parameters that must be considered. However, future investigations on transcription mechanisms in eukaryotic cells will generate more knowledge that will aid in the design of novel drugs targeting new proteins and DNA with enhanced affinities and effects on cancer cells. However, improvements in the pharmacological properties of transcription inhibitors can increase efficacy and potentially reduce toxicity and side effects.

In recent years, it has been established that the genomic information of a tumor will indicate specific treatments for each cancer patient. However, personalized therapy must take into account the fact that tumor cells evolve and genomes are heterogeneous. Therefore, the use of drugs with generalized effects will still be required. However, cancer stem cells are typically chemoresistant. Thus, additional studies on the effects of drugs that alter global transcription must be performed in cancer stem cells to determine which components of the basal transcription machinery preferentially affect cancer stem cells when depleted. It is important to consider whether the cancer type is susceptible to drugs that target the basal transcription machinery.

Although eukaryotic transcription machinery mechanisms still require additional investigation, novel discoveries will offer new targets for the treatment of cancer in the near future.

## Abbreviations

RNPI II, III: RNA polymerase I, II, III; TBP: TATA-binding protein; SL1: Selective factor 1; UBF: Upstream binding factor; TFII: Transcription factor II; SAGA: Spt–Ada–Gcn5–acetyltransferase; PIC: Pre-initiation complex; NER: Nucleotide excision repair; CTD: Carboxy-terminal domain; P-TEFb: Positive elongation factor b; CDK: Cyclin depending kinase; BRD4 3: Bromo domain containing protein 4, 3; HSF1: Heat shock factor 1; HCC: Hepatocellular carcinoma; JNK: June kinase; XPB XPD: Xeroderma pigmentosum,B or D; DSBs: Double strand breaks; MM: Multiple myeloma; ESC: Embryonic stem cells; AML: Acute myeloid leukemia; TGF: Transforming growth factor.

## Competing interest

The authors of this manuscript declare that they have no competing interests.

## Authors’ contributions

CV, GC and MZ wrote the manuscript. All authors read and approved the final manuscript.
